# Assessing the Prevalence Rate of Gangrene Among Patients With Peripheral Vascular Disease in a Tertiary Care Hospital in Central India

**DOI:** 10.7759/cureus.57038

**Published:** 2024-03-27

**Authors:** Abhilasha Bhargava, Chandrashekhar Mahakalkar, Shivani Kshirsagar, Simran Dhole

**Affiliations:** 1 General Surgery, Jawaharlal Nehru Medical College, Datta Meghe Institute of Higher Education & Research, Wardha, IND

**Keywords:** central india, clinical management, prevalence, gangrene, peripheral vascular disease

## Abstract

Background

Peripheral vascular disease (PVD) includes peripheral arterial disease (PAD) and venous disease. PAD is a chronic progressive atherosclerotic disease leading to partial or total peripheral vascular occlusion. PAD typically affects the abdominal aorta, iliac arteries, lower limbs, and occasionally the upper extremities. Assessing the prevalence of gangrene among PVD patients is crucial for understanding the burden of this condition and informing clinical management strategies. This study aimed to estimate the prevalence rate of gangrene among patients diagnosed with peripheral vascular disease.

Methods

This case-control study was conducted at the General Surgery department of Jawaharlal Nehru Medical College and Acharya Vinoba Bhave Rural Hospital, Wardha, India. Patients diagnosed with PVD who presented with gangrene of the lower limb were included. Detailed demographic data were collected, and clinical examinations and diagnostic tests were performed to assess the severity and extent of gangrene. Statistical analysis was conducted to estimate the prevalence of gangrene among PVD patients.

Results

Among the 100 participants, the age distribution ranged from 21 to over 70 years, with a mean age of 52.89. Gender distribution showed that 30% of the participants were female and 70% were male. The prevalence of gangrene among PVD patients was found to be 35%, with 65% patients not presenting with gangrene.

Conclusion

The findings of this study highlight the significant prevalence of gangrene among patients diagnosed with peripheral vascular disease. The implications of these findings for clinical practice and management strategies are discussed further, along with potential avenues for further research. The study provides valuable insights into the burden of gangrene among PVD patients. Early detection and appropriate management of PVD are crucial for preventing the development of gangrene and improving patient outcomes.

## Introduction

Peripheral vascular disease (PVD) refers to conditions affecting the blood vessels outside the heart and brain, primarily involving the arteries that supply blood to the limbs [1-2]. Atherosclerosis, characterized by plaque accumulation within the arterial walls, is the predominant cause of PVD. This narrowing and hardening of the arteries results in reduced blood flow to the extremities, leading to various clinical manifestations such as claudication, ischemic ulcers, and, in severe cases, gangrene [3-5].

Gangrene is a serious complication of PVD characterized by tissue death due to inadequate blood supply [3]. It can manifest as dry gangrene, resulting from gradual loss of blood flow without bacterial infection, or wet gangrene, which involves tissue necrosis accompanied by bacterial infection and decomposition. Gangrene poses a significant risk to limb viability and overall patient health, often necessitating aggressive management strategies, including surgical intervention and amputation [4].

The prevalence rate of gangrene among patients diagnosed with PVD serves as a critical indicator of disease burden and severity [5]. However, limited comprehensive data exist on the prevalence rate of gangrene in this population. Previous studies have reported varying prevalence rates, ranging from 20% to 60%, underscoring the need for further investigation to obtain accurate estimates and inform evidence-based interventions [6-9].

Understanding the prevalence of gangrene among PVD patients is crucial for optimizing patient care and resource allocation. Accurate estimation of gangrene prevalence can facilitate early detection, timely intervention, and preventive measures aimed at reducing the incidence of this debilitating complication. Therefore, this study aimed to systematically assess the prevalence rate of gangrene among patients diagnosed with peripheral vascular disease, contributing to a better understanding of the burden of this condition and informing clinical practice.

## Materials and methods

Study setting and design

The study was conducted as a case-control study at the General Surgery department of Jawaharlal Nehru Medical College and Acharya Vinoba Bhave Rural Hospital, Wardha, India, from 2022 to 2023.

Study population

The study included patients diagnosed with PVD who sought treatment at the General Surgery department. Inclusion criteria encompassed individuals of all ages and genders presenting with gangrene involving the lower limb. Patients with traumatic or infective causes of gangrene, venous obstruction, autoimmune vasculopathies, and those deemed unfit for inclusion were excluded.

Data collection

Patient selection for this study involved identifying individuals diagnosed with PVD who presented with gangrene of the lower limb. Informed consent was obtained from each participant before data collection. Detailed demographic data, including age, gender, and relevant medical history, were systematically recorded for each patient. A thorough clinical examination was conducted to assess the severity and extent of gangrene, including the presence of associated symptoms such as pain or numbness and complicating factors such as infection. The vascular status of the affected limb was evaluated through palpation of peripheral pulses and assessment of skin color and temperature. Diagnostic tests, including color Doppler imaging and ankle-brachial index measurements, were performed to confirm the diagnosis of peripheral vascular disease and assess the severity of vascular compromise. All data collected during the clinical evaluation and diagnostic tests were systematically recorded in a structured data collection form. The principal investigator reviewed and validated all collected data to ensure data accuracy and reliability. The prevalence of gangrene among patients with PVD was calculated based on the number of patients diagnosed with both conditions within the study population.

Statistical analysis

Statistical analysis was conducted to estimate the prevalence of gangrene among patients diagnosed with PVD. The prevalence was calculated as the proportion of patients diagnosed with both conditions relative to the total number of PVD patients. This calculation provided a point estimate of the prevalence, representing the percentage of PVD patients affected by gangrene. Statistical software IBM SPSS Statistics, version 23 (IBM Corp., Armonk, NY) was utilized to perform these calculations, facilitating efficient data management and analysis.

Ethical considerations

The Institutional Ethics Committee of Jawaharlal Nehru Medical College and Acharya Vinoba Bhave Rural Hospital approved the study protocol (approval no. DMIMS(DU)/IEC/Dec-2022/56). Informed consent was obtained from all participants before enrollment, and patient confidentiality was strictly maintained throughout the study.

## Results

Table 1 depicts the age distribution of the study participants, showing the frequency and percentage within each age category. The distribution ranged from 21 to over 70 years, with the mean age being 52.89 years and a standard deviation of 14.2 years. The median age fell to 54.5 years, with a range spanning from 45 to 63.25 years.

**Table 1:** Age distribution

Age (years)	Frequency	Percentage
21 to 30 years	11	11.00%
31 to 40 years	10	10.00%
41 to 50 years	19	19.00%
51 to 60 years	25	25.00%
61 to 70 years	30	30.00%
>70 years	5	5.00%
Mean ± SD	52.89 ± 14.2	
Median (25th-75th percentile)	54.5 (45-63.25)	
Range	21-83	

Table 2 presents the gender distribution of the study participants, detailing the frequency and percentage of females and males. Among the participants, 30% were female, while 70% were male, resulting in a total sample size of 100 individuals.

**Table 2:** Gender distribution

Gender	Frequency	Percentage
Female	30	30.00%
Male	70	70.00%
Total	100	100.00%

Table 3 displays the distribution of gangrene among the study participants, indicating the frequency and percentage of individuals with and without gangrene. Among the total sample of 100 participants, 65% did not have gangrene, while 35% presented with gangrene. Table 4 shows comorbidities and smoking status distribution among the participants.

**Table 3:** Gangrene distribution

Gangrene	Frequency	Percentage
No	65	65.00%
Yes	35	35.00%
Total	100	100.00%

**Table 4:** Comorbidities and smoking status distribution

Comorbidities/smoking status	Frequency	Percentage
Diabetes	45	45.00%
Hypertension	34	34.00%
Atherosclerosis	100	100.00%
Smoking - absent	37	37.00%
Smoking - present	63	63.00%
Total	279	279.00%

## Discussion

The prevalence of PVD presents a significant global health challenge, with potential complications such as gangrene leading to severe morbidity and mortality. In a recent study aimed at assessing the prevalence rate of gangrene among PVD patients, researchers shed light on the burden of this condition and its implications for clinical management strategies. Their findings revealed a considerable prevalence of gangrene among individuals diagnosed with PVD, with 35% of the participants presenting with this complication. This prevalence rate underscores the substantial impact of gangrene within the PVD population, emphasizing the urgent need for effective preventive measures and interventions. Similar prevalence rates have been reported in previous studies, reaffirming gangrene as a common and serious complication of PVD [10,11].

Moreover, the study delved into demographic patterns within the PVD population. It was observed that a larger proportion of male participants (70%) were affected compared to females (30%). This gender disparity aligns with the existing literature and may be attributed to various factors such as differences in lifestyle habits, genetic predispositions, and hormonal influences [12,13]. The wide age distribution among the study participants underscores the heterogeneous nature of individuals affected by PVD. This variability highlights the importance of personalized management approaches tailored to address specific needs and risk factors across different age groups [14,15].

Limitations

Despite the valuable insights that the study provides, several limitations need acknowledgment. First, the research was conducted at a single center, which may limit the generalizability of the findings to broader populations. Future multicenter studies involving larger and more diverse cohorts are warranted to validate and extend the findings. Additionally, the cross-sectional design of the study precludes causal inference. Longitudinal studies are needed to elucidate the temporal relationship between PVD and the development of gangrene. Also, its retrospective nature introduces potential data inaccuracies and exclusion criteria may have missed certain patient groups. These limitations point to avenues for future research to deepen the understanding of and improve clinical management strategies for PVD and its complications like gangrene.

## Conclusions

In conclusion, despite the limitations inherent in the study's single-center design, retrospective approach, and exclusion criteria, the findings shed light on the considerable prevalence of gangrene among individuals diagnosed with PVD. Moving forward, efforts to address these limitations through multicenter studies with larger, more diverse cohorts and prospective longitudinal designs are essential. Moreover, prioritizing interventions aimed at early detection and comprehensive management of PVD is crucial for preventing the development of gangrene and improving patient outcomes. By bridging these gaps in knowledge and practice, healthcare professionals can enhance care delivery for PVD patients, ultimately reducing the incidence and impact of gangrene. This study adds valuable insights to the existing literature on gangrene prevalence in the context of PVD, emphasizing the ongoing need for research and clinical efforts to mitigate the burden of this debilitating complication.

## References

[1] (2023). Peripheral vascular disease (PVD). https://www.yalemedicine.org/clinical-keywords/peripheral-vascular-disease.

[2] Zemaitis MR, Boll JM, Dreyer MA (2023). Peripheral arterial disease. StatPearls [Internet].

[3] (2023). Peripheral arterial disease (PAD). https://www.cdc.gov/heartdisease/PAD.htm.

[4] National Research Council (US) Committee on Diet and Health (1989). Atherosclerotic cardiovascular diseases. Diet and Health: Implications for Reducing Chronic Disease Risk.

[5] (2024). Peripheral vascular disease: what to know. https://www.medicalnewstoday.com/articles/322182.

[6] Buttolph A, Sapra A (2023). Gangrene. StatPearls [Internet].

[7] Bhargava A, Mahakalkar C, Kshirsagar S (2023). Understanding gangrene in the context of peripheral vascular disease: prevalence, etiology, and considerations for amputation-level determination. Cureus.

[8] Horváth L, Németh N, Fehér G, Kívés Z, Endrei D, Boncz I (2022). Epidemiology of peripheral artery disease: narrative review. Life (Basel).

[9] Sorensen MD, Krieger JN, Rivara FP, Broghammer JA, Klein MB, Mack CD, Wessells H (2009). Fournier's gangrene: population based epidemiology and outcomes. J Urol.

[10] DeRubertis BG, Pierce M, Ryer EJ, Trocciola S, Kent KC, Faries PL (2008). Reduced primary patency rate in diabetic patients after percutaneous intervention results from more frequent presentation with limb-threatening ischemia. J Vasc Surg.

[11] Hinchliffe RJ, Brownrigg JR, Andros G (2016). Effectiveness of revascularization of the ulcerated foot in patients with diabetes and peripheral artery disease: a systematic review. Diabetes Metab Res Rev.

[12] Liu J, Hong Y, D'Agostino RB Sr (2004). Predictive value for the Chinese population of the Framingham CHD risk assessment tool compared with the Chinese Multi-Provincial Cohort Study. JAMA.

[13] Norgren L, Hiatt WR, Dormandy JA, Nehler MR, Harris KA, Fowkes FG (2007). Inter-Society Consensus for the Management of Peripheral Arterial Disease (TASC II). J Vasc Surg.

[14] Fowkes FGR, Rudan D, Rudan I (2013). Comparison of global estimates of prevalence and risk factors for peripheral artery disease in 2000 and 2010: a systematic review and analysis. Lancet.

[15] Gerhard-Herman MD, Gornik HL, Barrett C (2017). 2016 AHA/ACC guideline on the management of patients with lower extremity peripheral artery disease: executive summary: a report of the American College of Cardiology/American Heart Association Task Force on Clinical Practice Guidelines. Circulation.

